# Risk-to-befit ratios of consecutive antidepressants for heavy menstrual bleeding in young women with bipolar disorder or major depressive disorder

**DOI:** 10.3389/fpsyt.2022.1012644

**Published:** 2022-10-28

**Authors:** Chuanjun Zhuo, Guangdong Chen, Chongguang Lin, Jing Ping, Jingjing Zhu, Lina Wang, Shili Jin, Chuanxin Liu, Qiuyu Zhang, Lei Yang, Qianchen Li, Chunhua Zhou, Langlang Cheng, Hongjun Tian, Xueqin Song

**Affiliations:** ^1^Department of Psychiatry, Wenzhou Seventh Peoples Hospital, Wenzhou, China; ^2^Key Laboratory of Multiple Organs Damage in Patients With Metal Disorder, Tianjin Fourth Center Hospital, Nankai University Affiliated Tianjin Fourth Center Hospital, Tianjin, China; ^3^Department of Psychiatry, Tianjin Fourth Center Hospital, Nankai University Affiliated Tianjin Fourth Center Hospital, Tianjin, China; ^4^Department of Psychiatry, The First Affiliated Hospital of Zhengzhou University, Zhengzhou, China; ^5^Laboratory of Psychiatric-Neuroimaging-Genetic and Comorbidity, Tianjin Anding Hospital, Tianjin Mental Health Center of Tianjin Medical University, Tianjin, China; ^6^Inpatient Department, Shandong Daizhuang Hospital, Jining, China; ^7^College of Mental Disorder, Jining Medical University, Jining, China; ^8^Department of Pharmacology, The First Hospital of Hebei Medical University, Shijiazhuang, China

**Keywords:** heavy menstrual bleeding, antidepressant agents, valproate, bipolar disorder, major depressive disorder, risk factors

## Abstract

The occurrence of heavy menstrual bleeding (HMB) induced by pharmacological agents has been reported in young adult women. This study aimed to investigate a possible association between the occurrence rates of HMB and different treatment methods such as antidepressant agents alone and in combination with other pharmacological agents. The examined cohort included young women (age 18–35 years, *n* = 1,949) with bipolar disorder (BP) or major depressive disorder (MDD). Menstruation history for 24 months was recorded and evaluated according to pictorial blood loss assessment charts of HMB. Multivariate analyses were conducted to determine odds ratios (ORs) and 95% confidence intervals. The examined antidepressant agents had varying ORs for patients with BP vs. those with MDD. For example, the ORs of venlafaxine-induced HMB were 5.27 and 4.58 for patients with BP and MDD, respectively; duloxetine-induced HMB, 4.72 and 3.98; mirtazapine-induced HMB, 3.26 and 2.39; fluvoxamine-induced HMB, 3.11 and 2.08; fluoxetine-induced HMB, 2.45 and 1.13; citalopram-induced HMB, 2.03 and 1.25; escitalopram-induced HMB, 1.85 and 1.99; agomelatine-induced HMB, 1.45 and 2.97; paroxetine-induced HMB, 1.19 and 1.75; sertraline-induced HMB, 0.88 and 1.13; reboxetine-induced HMB, 0.45 and 0.45; and bupropion-induced HMB, 0.33 and 0.37, in each case. However, when antidepressant agents were combined with valproate, the OR of HMB greatly increased, with distinct profiles observed for patients with BP vs. those with MDD. For example, the ORs of HMB induced by venlafaxine combined with valproate were 8.48 and 6.70 for patients with BP and MDD, respectively; for duloxetine, 5.40 and 4.40; mirtazapine, 5.67 and 3.73; fluvoxamine, 5.27 and 3.37; fluoxetine, 3.69 and 4.30; citalopram, 5.88 and 3.46; escitalopram, 6.00 and 7.55; agomelatine, 4.26 and 5.65; paroxetine, 5.24 and 3.25; sertraline, 4.97 and 5.11; reboxetine, 3.54 and 2.19; and bupropion, 4.85 and 3.46, in each case. In conclusion, some antidepressant agents exhibited potential risks of inducing HMB. Therefore, a combined prescription of antidepressant agents and valproate should be carefully considered for young women with HMB.

## Introduction

Serious mood disorders such as bipolar disorder (BP) and major depressive disorder (MDD) (1) have high prevalence among young women (aged 18–35 years) (2–9). Typically, these disorders are treated using mood stabilizers [not a true pharmacological category (10)] and antidepressants (11–17). However, these treatments could result in adverse secondary effects (18, 19), including menstrual dysfunction (20–30) (e.g., hypomenorrhea/amenorrhea) and heavy menstrual bleeding (HMB) (31).

Antidepressant agents reportedly induce HMB, which can cause pain and distress in patients (32–34). Thus, the US Food and Drug Administration has stated that psychiatric drug labeling warning guidelines are undergoing revision to include the risk of HMB as a possible side effect. Currently, however, patient information leaflets for venlafaxine, sertraline, and fluoxetine, which are commonly prescribed antidepressant agents, do mention bleeding as an aggravating side effect.

HMB, defined as excessive regular or irregular menstrual bleeding (>80 mL/cycle) (35), affects 4% of women without organic pathology (36, 37). Antidepressant agents and mood stabilizers have been associated with hemorrhage/bleeding (1, 38–40). In female patients with severe mood disorders, hemorrhage/bleeding mainly presents as HMB (10, 41), which can lead to further psychiatric deterioration (42–46). HMB in women has also been associated with metabolic disorders of blood glucose, lipids, and reproductive hormones (42–50), all of which can be induced or exacerbated by antidepressant drugs (42, 43, 46–61). Moreover, HMB can impair cognitive ability (10, 41–61), which may also be directly affected by mental illness and its treatments (10, 41–62). Additionally, HMB may compromise a patient's reproductive potential (10, 42–62). Hence, a multitude of negatively interacting factors related to HMB exists, which could worsen the prognosis of young women with serious mood disorders.

Hence, to address the aforementioned unresolved questions regarding HMB risk in young women with BP and MDD, this retrospective multi-hospital study aimed to determine HMB occurrence rates and identify possible associations between the occurrence rates and use of various antidepressant agents with and without valproate.

## Methods

### Study design and participants

In this retrospective cohort study, convenience sampling was used to recruit young adult females being treated by senior psychiatrists (*n* = 514) in outpatient departments. To help assess HMB, 10 gynecologists were invited to contribute to this study. The physician recruitment period lasted 2 months (March 1–31, 2022). Recruited doctors furnished detailed information regarding the patients, including their sociodemographic characteristics diagnosis; history of menstrual cycle timing from January 1, 2019 through February 28, 2022; HBM incidence; and antidepressant agent categories and dosages. Informed consent forms were signed by patients and their guardians before data collection. The ethics committee of Tianjin Fourth Center Hospital approved this study (No. ZC-R-0001).

The inclusion criteria were (1) females aged 18–35 years experiencing their first episode of BP or MDD, (2) an understanding of one's own mental illness and treatment methods, (3) normal memory ability (to ensure recall of periods over the previous 24 months), (4) available medical records to confirm an absence of neurological or physical disease comorbidity, any history of menstrual dysfunction, and pharmacological therapies administered within the previous 24 months, and (5) willingness to participate in this study as a volunteer and provide detailed personal sociodemographic information. The exclusion criteria were (1) unwillingness to participate in the study; (2) inability to recall menstrual history for the previous 24 months; (3) history of pregnancy and/or abortion within the previous 24 months [with pregnancy tests conducted (63)]; (4) history of neurological illness, physical disease, or substance abuse history within the previous 24 months; (5) any other mental disorder comorbidity; (6) no major stressful life events within the previous 24 months; (7) having no female family member/guardian available to assist with the collection of information concerning the patient's illness, menstrual status, HMB status, and other needed information; (8) abnormal bleeding and coagulation time of participants at the specified time point; and (9) within in the menstrual cycle in the patients who at the time point participated in the present study.

### Procedures

#### Data collection

Clinical information was collected during an insurance settlement period in China that extended over 3 months. For this period, each participating physician collated the following information for each participating patient: category of mood illness, total number of menstrual cycles within the previous 24 months, HMB incidence rates within the previous 24 months, and total cumulative medication dosage within the previous 24 months. Each patient's medical record was consulted to confirm the medication dosage and an absence of neurological and physical disease history within the previous 24 months. Cumulative use of antidepressants, mood stabilizers, and anxiolytic/sleep aides during the previous 24 months were converted to chlorpromazine (64), fluoxetine (65), sodium valproate (66), and diazepam equivalent data (67).

#### Instruments

BP and MDD definitions were adopted from the Diagnostic and Statistical Manual of Mental Disorders–Edition IV (DSM-IV) (62) and Structured Clinical Interview for DSM-IV Axis I Disorders (68). Mood illness etiology was described based on core symptoms. Patient insight was confirmed using the Birchwood Insight Scale (69) and Beck Cognitive Insight Scale self-report measures (63). Normal memory function was confirmed using a Chinese version of the Wechsler Memory Scale (4th edition) (70).

The Pictorial Blood loss Assessment Chart (PBAC) (71) was used to assess HMB. However, although PBAC is currently used extensively to assess HMB, its validity regarding previous HMB status remains to be verified. Because of the retrospective design of this study, gynecologists illustrated the HBM of patients according to PBAC. The gynecologists also provided guidance regarding information collection, including specific information on the presence of blood clots on sanitary napkins, how many sanitary napkins were used in one menstrual period, and how to calculate menstrual bleeding. Although previous studies have used more accurate HMB diagnostic criteria, our large sample size precluded such a highly complex procedure from being adopted in the present study. The use of PBAC also represents a limitation of the present study; however, it provided useful information.

### Statistical analysis

Statistical analyses were performed using the SAS statistical software (version 9.3, SAS Institute, Cary, NC, USA). Continuous variable data exhibiting normal distribution are presented as mean ± standard deviation. Data were compared across groups and within groups over time using analyses of variance (ANOVA) and repeated-measures ANOVA, respectively. Categorical variable data are presented as numbers and percentages. Associations of clinicodemographic characteristics with HMB incidence were evaluated using univariate and multivariate logistic regression models and are expressed as odds ratios (ORs) and 95% confidence intervals (CIs) in the overall sample and according to the diagnosis group. Multivariate logistic models were first developed by adjusting for factors that were significant in the univariate analyses (*p* < 0.01). The final multivariate models were limited to risk factors or confounders that were statistically significant (66, 67, 72–80).

## Results

### Patient and study characteristics

In total, 1,949 female participants, including 855 with BP and 1,094 with MDD, were recruited. Data regarding patient age, education, and illness duration are presented in Table 1; no significant differences were observed between the BP and MDD groups. Antidepressant treatment use and cumulative dosages in the BP and MDD groups are presented in Table 2. Data regarding HMB awareness as indicated on the HMB self-report scale are summarized in Table 3. While most participating patients indicated that HMB had negative effects on their illness progression and quality of life, few patients had been informed that HMB was a potential secondary adverse reaction to psychiatric medications.

**Table 1:** Sociodemographic characteristics by group diagnosis and total population included.

**Variable**	**BP**	**MDD**	**All**
	***N* = 855**	***N* = 1,094**	***N* = 1,904**
Education, >12 years, *n* (%)	557 (65.1)	556 (53.0)	1,113 (57.1)
Age of illness onset, years, mean ± SD	27.9 ± 3.7	27.6 ± 3.9	27.7 ± 3.8
Total illness duration, months., mean ± SD	45.4 ± 4.0	47.2 ± 6.3	46.5 ± 8.3

BP, bipolar disorder; MDD, major depressive disorder; SD, standard deviation.

**Table 2:** Therapeutic agents and mean cumulative dosages.

**Variable**	**BP**	**MDD**	**All**
	***N* = 855**	***N* = 1,094**	***N* = 1,949**
Cumulative dosage of antidepressant agents (equivalent dose of fluoxetine, mg)	8,960.5 ± 8,558.7	66,350.9 ± 10,258.4	42,333.8 ± 9,876.9
Cumulative dosage of sodium valproate (mg)	576,820.6 ± 11,835.26	333,489.0 ± 9,925.8	435,887.5 ± 109,258.4
Cumulative dosage of lithium (mg)	432,587.5 ± 12,859.6	333,693.7 ± 2,456.2	398,523.4 ± 11,563.2
Cumulative dosage of antipsychotic agents (mg)	285,369.5 ± 12,735.9	222,385.5 ± 8,956.9	258,746.5 ± 7,532.6
Cumulative dosage of sedative agents (mg)	5,897.6 ± 697.5	3,365.0 ± 951.9	4,115.5 ± 753.4

BP, bipolar disorder; MDD, major depressive disorder.

**Table 3:** HMB occurrence rates according to the treatment for each diagnosed group and total sample population.

**Variable**		**BP**	**MDD**	**All**
		***N* = 267**	***N* = 218**	***N* = 485**
Venlafaxine-associated HMB	No	1	3	4
	Yes	13	8	21
Duloxetine-associated HMB	No	1	2	3
	Yes	6	8	14
Mirtazapine-associated HMB	No	2	2	4
	Yes	6	9	15
Fluvoxamine-associated HMB	No	5	3	8
	Yes	8	5	13
Fluoxetine-associated HMB	No	3	4	7
	Yes	6	7	13
Citalopram-associated HMB	No	2	1	3
	Yes	5	3	8
Escitalopram-associated HMB	No	1	5	6
	Yes	3	7	10
Agomelatine-associated HMB	No	1	2	3
	Yes	2	3	5
Paroxetine-associated HMB	No	5	3	8
	Yes	12	4	16
Sertraline-associated HMB	No	3	4	7
	Yes	4	6	10
Reboxetine-associated HMB	No	1	1	2
	Yes	1	1	2
Bupropion-associated HMB	No	2	2	4
	Yes	2	1	3
Venlafaxine + valproate-associated HMB	No	2	5	7
	Yes	32	22	54
Duloxetine + valproate-associated HMB	No	7	2	9
	Yes	16	10	26
Mirtazapine + valproate-associated HMB	No	4	5	9
	Yes	7	13	20
Fluvoxamine + valproate-associated HMB	No	8	3	11
	Yes	16	10	26
Fluoxetine + valproate-associated HMB	No	6	6	12
	Yes	14	9	23
Citalopram + valproate-associated HMB	No	3	3	6
	Yes	12	6	18
Escitalopram + valproate-associated HMB	No	2	1	3
	Yes	7	3	10
Agomelatine + valproate-associated HMB	No	1	1	2
	Yes	3	5	8
Paroxetine + valproate-associated HMB	No	6	5	11
	Yes	9	7	16
Sertraline + valproate-associated HMB	No	2	1	3
	Yes	4	1	5
Reboxetine + valproate-associated HMB	No	2	1	3
	Yes	1	1	2
Bupropion + valproate-associated HMB	No	5	3	8
	Yes	3	1	4

HMB, heavy menstrual bleeding; BP, bipolar disorder; MDD, major depressive disorder.

### HBM occurrence rates and associated factors

Among the 1,949 participants, 653 (33.50%) experienced HMB, and the average frequency of HMB within the previous 24 months was 3.0 ± 0.4 menstrual cycles. Among the 653 participants, 485 (24.88%) received a single antidepressant agent or a combination of an antidepressant agent and valproate to alleviate their diseases. Among the 485 participants, 267 (55.05%) had BP and 218 (44.95%) had MDD. Unexpectedly, only 1.1% of our patients considered the occurrence of HBM to be associated with the prescribed treatment agents. The HMB occurrence rates according to diagnosis were 30.53% (267/855) and 19.92% (218/1,094) for BP and MDD, respectively. These data indicate a 1.53-fold higher prevalence of HMB in patients with BP. The univariate and multivariate logistic regression analyses demonstrated that high dosages of antidepressant agents were not strong risk factors for HMB in either group [BP: OR, 4.36 (95% CI: 0.89–9.71); MDD: OR, 2.28 (95% CI: 0.74–4.84)].

However, the univariate and multivariate logistic regression analyses revealed that different antidepressant agents had varying ORs in patients with BP vs. those with MDD (Tables 4, 5). For example, in decreasing order, the ORs of venlafaxine-induced HMB were 5.27 (95% CI: 2.88–8.69) and 4.58 (95% CI: 2.31–7.56) in patients BP and MDD, respectively; for duloxetine, 4.72 (95% CI: 1.59–8.13) and 3.98 (95% CI: 2.07–6.03); for mirtazapine, 3.26 (95% CI: 2.00–9.67) and 2.39 (95% CI: 1.13–5.00); for fluvoxamine, 3.11 (95% CI: 1.71–9.18) and 2.08 (95% CI: 1.10–5.57); for fluoxetine, 2.45 (95% CI: 1.12–5.80) and 1.13 (95% CI: 1.00–3.09); for citalopram, 2.03 (95% CI: 1.07–8.71) and 1.25 (95% CI: 1.00–8.13); for escitalopram, 1.85 (95% CI: 1.23–4.17) and 1.99 (95% CI: 1.05–9.65); for agomelatine, 1.45 (95% CI: 1.00–3.40) and 2.97 (95% CI: 1.97–23.22); for paroxetine, 1.19 (95% CI: 1.02–3.40) and 1.75 (95% CI: 1.25–4.20); for sertraline, 0.88 (95% CI: 0.44–1.22) and 1.13 (95% CI: 0.45–2.64); for reboxetine, 0.45 (95% CI: 0.12–0.88) and 0.45 (95% CI: 0.14–0.92); and for bupropion, 0.33 (95% CI: 0.12–0.67) and 0.37 (95% CI: 0.58–0.73), respectively, in each case. Thus, some antidepressant agents exhibited a potential risk for inducing HMB (Tables 4, 5).

**Table 4:** Univariate analysis results for each group according to various treatment methods.

		**BP**	**MDD**	**All**
**Treatment method**		**OR (95% CI)**	**OR (95% CI)**	**OR (95% CI)**
Education	≤ 12 years	1.0	1.0	1.0
	>12 years	1.00 (0.82–1.99)	0.88 (0.50–2.47)	0.94 (0.64–1.14)
Illness onset age, < 20 years		6.23 (4.51–10.23)	4.52 (3.25–8.59)	3.97 (1.45–5.23)
Total illness duration, mos.		2.87 (0.94–4.00)	1.95 (0.44–3.61)	2.69 (0.37–8.92)
Venlafaxine-associated HMB	No	1.0	1.0	1.0
	Yes	5.53 (2.98–10.25)	4.89 (2.99–8.33)	5.23 (3.89–11.85)
Duloxetine-associated HMB	No	1.0	1.0	1.0
	Yes	5.12 (1.77–9.25)	4.00 (2.22–6.88)	4.99 (2.77–11.22)
Mirtazapine-associated HMB	No	1.0	1.0	1.0
	Yes	4.02 (2.44–9.99)	3.12 (1.27–5.44)	3.02 (2.10–5.36)
Fluvoxamine-associated HMB	No	1.0	1.0	1.0
	Yes	3.48 (2.22–9.47)	2.75 (1.70–6.78)	2.97 (1.99–5.19)
Fluoxetine-associated HMB	No	1.0	1.0	1.0
	Yes	2.66 (1.40–6.98)	1.43 (1.27–5.11)	1.97 (1.58–4.00)
Citalopram-associated HMB	No	1.0	1.0	1.0
	Yes	2.16 (1.42–9.17)	1.42 (1.07–9.25)	1.85 (1.91–7.35)
Escitalopram-associated HMB	No	1.0	1.0	1.0
	Yes	2.04 (1.29–5.19)	2.19 (1.73–8.53)	2.77 (1.69–9.60)
Agomelatine-associated HMB	No	1.0	1.0	1.0
	Yes	1.85 (1.40–3.99)	3.00 (2.00–25.10)	2.07 (1.19–12.58)
Paroxetine-associated HMB	No	1.0	1.0	1.0
	Yes	1.39 (1.11–3.88)	1.79 (1.44–4.35)	1.88 (1.45–4.39)
Sertraline-associated HMB	No	1.0	1.0	1.0
	Yes	0.88 (0.44–1.22)	1.13 (0.45–2.64)	1.07 (0.45–1.97)
Reboxetine-associated HMB	No	1.0	1.0	1.0
	Yes	0.56 (0.22–0.98)	0.57 (0.34–0.85)	0.66 (0.18–0.77)
Bupropion-associated HMB	No	1.0	1.0	1.0
	Yes	0.45 (0.22–0.87)	0.45 (0.69–0.93)	0.32 (0.52–0.60)
Cumulative dosage of sodium valproate	No	1.0	1.0	1.0
	Yes	8.52 (3.94–11.80)	6.78 (3.44–10.13)	7.21 (3.60–9.28)
Cumulative dosage of lithium	No	1.0	1.0	1.0
	Yes	3.21 (1.36–9.74)	2.79 (1.19–5.87)	2.11 (1.98–3.76)
Cumulative dosage of antipsychotic agents	No	1.0	1.0	1.0
	Yes	0.75 (0.33–1.28)	0.35 (0.55–0.97)	0.66 (0.35–0.83)
Venlafaxine + valproate-associated HMB	No	1.0	1.0	1.0
	Yes	7.36 (2.33–11.22)	6.33 (2.00–13.97)	6.66 (1.88–10.99)
Duloxetine + valproate-associated HMB	No	1.0	1.0	1.0
	Yes	5.57 (3.11–9.44)	4.30 (2.50–8.55)	4.76 (2.09–12.97)
Mirtazapine + valproate-associated HMB	No	1.0	1.0	1.0
	Yes	6.11 (3.25–11.30)	4.44 (2.88–17.25)	5.48 (2.30–15.23)
Fluvoxamine + valproate-associated HMB	No	1.0	1.0	1.0
	Yes	7.11 (4.89–15.43)	8.88 (5.99–14.76)	8.02 (2.14–18.25)
Fluoxetine + valproate-associated HMB	No	1.0	1.0	1.0
	Yes	3.49 (1.63–7.82)	4.54 (1.88–9.77)	4.06 (1.44–12.41)
Citalopram + valproate-associated HMB	No	1.0	1.0	1.0
	Yes	3.99 (2.45–7.88)	4.25 (2.33–10.22)	3.98 (1.66–13.64)
Escitalopram + valproate-associated HMB	No	1.0	1.0	1.0
	Yes	4.48 (2.13–9.99)	6.25 (2.44–10.52)	5.64 (1.49–15.96)
Agomelatine + valproate-associated HMB	No	1.0	1.0	1.0
	Yes	4.70 (1.33–9.44)	3.88 (2.54–9.77)	4.06 (2.30–12.56)
Paroxetine + valproate-associated HMB	No	1.0	1.0	1.0
	Yes	3.26 (1.47–8.69)	2.59 (1.20–5.77)	2.88 (2.58–10.47)
Sertraline + valproate-associated HMB	No	1.0	1.0	1.0
	Yes	3.99 (2.44–9.66)	4.25 (1.99–10.00)	4.03 (1.44–15.22)
Reboxetine + valproate-associated HMB	No	1.0	1.0	1.0
	Yes	6.59 (2.55–12.47)	4.56 (2.34–10.55)	5.46 (2.05–15.47)
Bupropion + valproate-associated HMB	No	1.0	1.0	1.0
	Yes	4.22 (1.58–11.46)	3.66 (1.26–9.77)	3.97 (1.17–11.22)

OR, odds ratio; CI, confidence interval; BP, bipolar disorder; MDD, major depressive disorder; HMB, heavy menstrual bleeding.

**Table 5:** Multivariate analysis results of the association of HMB with various treatment methods.

**Risk Factor**	**BP**		**MDD**		**All**	
	**OR (95% CI)**	***P***	**OR (95% CI)**	***P***	**OR (95% CI)**	***P***
Venlafaxine-associated HMB	1.0 5.27 (2.88–8.69)	< 0.0001	1.0 4.58 (2.31–7.56)	< 0.0001	1.0 4.79 (3.30–8.20)	< 0.0001
Duloxetine-associated HMB	1.0 4.72 (1.59–8.13)	< 0.0001	1.0 3.98 (2.07–6.03)	< 0.0001	1.0 4.88 (2.45–9.58)	< 0.0001
Mirtazapine-associated HMB	1.0 3.26 (2.00–9.67)	< 0.0001	1.0 2.39 (1.13–5.00)	< 0.0001	1.0 2.87 (1.96–4.89)	< 0.0001
Fluvoxamine-associated HMB	1.0 3.11 (1.71–9.18)	< 0.0001	1.0 2.08 (1.10–5.57)	< 0.0001	1.0 2.58 (1.45–5.78)	< 0.0001
Fluoxetine-associated HMB	1.0 2.45 (1.12–5.80)	< 0.0001	1.0 1.13 (1.00–3.09)	< 0.0001	1.0 1.63 (1.32–3.85)	< 0.0001
Citalopram-associated HMB	1.0 2.03 (1.07–8.71)	< 0.0001	1.0 1.25 (1.00–8.13)	< 0.0001	1.0 1.55 (1.12–9.23)	< 0.0001
Escitalopram-associated HMB	1.0 1.85 (1.23–4.17)	< 0.0001	1.0 1.99 (1.05–9.65)	< 0.0001	1.0 2.03 (1.17–8.46)	< 0.0001
Agomelatine-associated HMB	1.0 1.45 (1.00–3.40)	< 0.0001	1.0 2.97 (1.97–23.22)	< 0.0001	1.0 1.89 (1.20–9.53)	< 0.0001
Paroxetine-associated HMB	1.0 1.19 (1.02–3.40)	< 0.0001	1.0 1.75 (1.25–4.20)	< 0.0001	1.0 1.44 (1.20–4.88)	< 0.0001
Reboxetine-associated HMB	1.0 0.45 (0.12–0.88)	< 0.0001	1.0 0.45 (0.14–0.92)	< 0.0001	1.0 0.43 (0.18–0.99)	< 0.0001
Bupropion-associated HMB	1.0 0.33 (0.12–0.67)	< 0.0001	1.0 0.37 (0.58–0.73)	< 0.0001	1.0 0.35 (0.59–0.67)	< 0.0001
Cumulative dosage of sodium valproate	1.0 8.27 (4.85–15.27)	< 0.0001	1.0 7.45 (3.69–10.33)	< 0.0001	1.0 7.84 (2.85–12.36)	< 0.0001
Venlafaxine + valproate-associated HMB	1.0 8.48 (2.44–14.17)	< 0.0001	1.0 6.70 (2.58–12.54)	< 0.0001	1.0 7.12 (3.74–15.40)	< 0.0001
Duloxetine + valproate-associated HMB	1.0 5.40 (2.24–10.07)	< 0.0001	1.0 4.40 (2.50–10.24)	< 0.0001	1.0 4.75 (2.71–10.87)	< 0.0001
Mirtazapine + valproate-associated HMB	1.0 5.67 (4.11–11.00)	< 0.0001	1.0 3.73 (1.22–7.74)	< 0.0001	1.0 4.29 (1.58–9.99)	< 0.0001
Fluvoxamine + valproate-associated HMB	1.0 5.27 (2.00–7.89)	< 0.0001	1.0 3.37 (1.29–8.70)	< 0.0001	1.0 4.09 (2.22–14.86)	< 0.0001
Fluoxetine + valproate-associated HMB	1.0 3.69 (2.10–10.25)	< 0.0001	1.0 4.30 (1.99–9.50)	< 0.0001	1.0 3.99 (2.10–9.55)	< 0.0001
Citalopram + valproate-associated HMB	1.0 5.88 (2.66–10.25)	< 0.0001	1.0 3.46 (2.07–6.44)	< 0.0001	1.0 3.87 (2.14–10.46)	< 0.0001
Escitalopram + valproate-associated HMB	1.0 6.00 (2.19–9.26)	< 0.0001	1.0 7.55 (3.44–12.11)	< 0.0001	1.0 6.93 (3.33–10.47)	< 0.0001
Agomelatine + valproate-associated HMB	1.0 4.26 (2.19–7.81)	< 0.0001	1.0 5.65 (3.24–10.33)	< 0.0001	1.0 4.99 (2.99–11.11)	< 0.0001
Paroxetine + valproate-associated HMB	1.0 5.24 (3.66–12.57)	< 0.0001	1.0 3.25 (1.50–9.44)	< 0.0001	1.0 4.36 (2.71–13.33)	< 0.0001
Sertraline + valproate-associated HMB	1.0 4.97 (2.32–9.60)	< 0.0001	1.0 5.11 (1.45–12.54)	< 0.0001	1.0 4.62 (2.37–10.99)	< 0.0001
Reboxetine + valproate-associated HMB	1.0 3.54 (1.66–8.10)	< 0.0001	1.0 2.19 (1.39–10.77)	< 0.0001	1.0 3.22 (2.14–16.46)	< 0.0001
Bupropion + valproate-associated HMB	1.0 4.85 (1.19–9.89)	< 0.0001	1.0 3.41 (2.45–13.48)	< 0.0001	1.0 4.22 (1.10–15.77)	< 0.0001

OR, odds ratio; CI, confidence interval; BP, bipolar disorder; MDD, major depressive disorder; HMB, heavy menstrual bleeding.

More importantly, both the univariate and multivariate logistic regression analyses demonstrated that when different antidepressant agents are combined with valproate, the ORs for HMB varied in patients with BP vs. those with MDD (Tables 4, 5). For example, in decreasing order, the ORs of valproate-induced HMB with venlafaxine were 8.48 (95% CI: 2.44–14.17) and 6.70 (95% CI: 2.58–12.54) in patients with BP and MDD, respectively; for valproate-induced HMB with duloxetine, 5.40 (95% CI: 2.24–10.07) and 4.40 (95% CI: 2.50–10.24); with mirtazapine, 5.67 (95% CI: 4.11–11.00) and 3.73 (95% CI: 1.22–7.74); with fluvoxamine, 5.27 (95% CI: 2.00–7.89) and 3.37 (95% CI: 1.29–8.70); with fluoxetine, 3.69 (95% CI: 2.10–10.25) and 4.30 (95% CI: 1.99–9.50); with citalopram, 5.88 (95% CI: 2.66–10.25) and 3.46 (95% CI: 2.07–6.44); with escitalopram, 6.00 (95% CI: 2.19–9.26) and 7.55 (95% CI: 3.44–12.11); with agomelatine, 4.26 (95% CI: 2.19–7.81) and 5.65 (95% CI: 3.24–10.33); with paroxetine, 5.24 (95% CI: 3.66–12.57) and 3.25 (95% CI: 1.50–9.44); with sertraline, 4.97 (95% CI: 2.32–9.60) and 5.11 (95% CI: 1.45–12.54); with reboxetine, 3.54 (95% CI: 1.66–8.10) and 2.19 (95% CI: 1.39–10.77); and with bupropion, 4.85 (95% CI: 1.19–9.89) and 3.46 (95% CI: 2.45–13.48), respectively, in each case. Thus, certain antidepressant agents exhibited a potential risk for inducing HMB (Tables 4, 5). Furthermore, the use of an antidepressant agent with valproate resulted in an increased risk for HMB in patients with BP and MDD. Altogether, these data indicate that in clinical practice, doctors should pay greater attention to monitoring HMB when treating patients with BP or MDD.

## Discussion

The our results indicate that the occurrence rates for HMB among young adult females with mood disorders is high, especially among those with BP and MDD (24.88%, more than 6-fold the rate for healthy young adult female controls). Unexpectedly, only 1.1% adult women considered HMB to be associated with their treatment agents. Additionally, the present data demonstrate that antidepressant agents represent a risk factor for HMB in young adult females. When antidepressants were prescribed as monotherapy for patients with BP or MDD, the risk for HMB in decreasing order was venlafaxine > duloxetine > mirtazapine > fluvoxamine > citalopram > escitalopram > agomelatine. Furthermore, a protective effect toward HMB was consistently and unexpectedly observed for reboxetine and bupropion among patients with BP or MDD.

Notably, and more significantly, when various antidepressant agents were administered in combination with valproate, the OR of HMB further increased, although it exhibited an inconsistent pattern among patients with BP vs. those with MDD. For example, the risk for HMB in decreasing order according to the combined treatment regimen in patients with BP was venlafaxine > escitalopram > citalopram > mirtazapine > duloxetine > fluvoxamine > paroxetine > sertraline > bupropion > agomelatine > fluoxetine > reboxetine. Contrastingly, the risk for HMB in decreasing order in patients with MDD was escitalopram > venlafaxine > agomelatine > sertraline > duloxetine > fluoxetine > mirtazapine > citalopram > bupropion > fluvoxamine > paroxetine > reboxetine.

Studies have reported that valproate can increase the risk of HMB (51–53). However, to the best of our knowledge, the present data represent the first evidence that young adult females with mood disorders need to be monitored for HMB, especially when an antidepressant agent is prescribed with valproate. However, our present data demonstrate that the OR of HMB for treatment agents combined with valproate exhibited a consistent trend among patients with BP, but the pattern was inconsistent among patients with MDD. Based on these data, we recommend conducting further studies to characterize the mechanisms of this phenomenon. In this study, HMB was more prevalent in patients with BP than those with MDD. The reasons for this difference are unknown, thus further studies are warranted. Our patients had higher rates of menstrual dysfunction than previously reported (81–83).

Regarding the possible mechanisms underlying antidepressant-induced HMB, some studies reported that antidepressant-induced menstrual dysfunction could be attributed to drug-induced disruption of the hypothalamic-pituitary-ovarian (H-P-O) axis. This alters the estrogen and progesterone cycles that regulate menstruation (41, 62). HMB may occur after several months of amenorrhea/oligomenorrhea, during which the endometrium cannot disintegrate normally, which could result in endometrial hyperplasia (41, 62). Thus, HMB may reflect a disorder of estrogen and progesterone secretion independent of hyper-prolactin. In women with BP, valproate reportedly induces hyperandrogenism, which leads to oligomenorrhea, consistent with an H-P-O disturbance (25, 84). Pharmacotherapeutic-induced hyper-prolactin reflects a cryptorrhea phenomenon, the effects of which should be elucidated in a prospective cohort study. Psychiatric medications have secondary effects on the hemic system (54, 85–87) and thus can cause or exacerbate coagulation disorders and abnormal bleeding, which can result in HMB (88). Although the precise mechanisms underlying these drug effects are unknown, physicians should screen female psychiatric patients for HMB during patient monitoring. To the best of our knowledge, there is a dearth of studies reporting the association of antidepressant agents in other races. However, labels and instructions of many antidepressants state that antidepressant agents can cause HMB. Hence, in the future, multicenter cohort studies (including different racial participants—both patients and healthy controls) should be conducted to clarify the relationship between antidepressant agents and HMB and explore the mechanisms underlying antidepressants causing HMB.

This study had several limitations. First, this retrospective study employed PBAC to assess HMB history. The study design was inferior to a prospective study design. Hence, in the future, we plan to conduct a prospective study to validate our present findings. Second, the validity of PBAC for assessing menstrual bleeding according to the previous month's menstrual bleeding pattern remains to be confirmed. To partly address this, the participating patients in this study were confirmed to possess good memory according to the Wechsler memory scale, and the status of their last menstrual bleeding was used as a reference standard. Third, most of our patients in this study used aripiprazole and traditional Chinese medicines to treat menstrual dysfunction. However, none of the samples had normalized menstrual function during the study. Although these factors were regressed, it is possible that the results obtained from the patients examined in this study may not be generalizable to all patients with BP or MDD, because some of the patients' menstrual dysfunction could have been normalized by the aripiprazole and traditional Chinese medicines.

## Conclusion

This study yielded three pivotal clinical reference findings: (1) the risk for HMB in young adult women is substantial; (2) antidepressant categories are highly related to HMB occurrence. Thus, healthcare providers should screen female patients for HMB and adjust treatment plans accordingly; and (3) the use of valproate can greatly increase the OR of HMB in both patients with BP and MDD, although different antidepressant agents combined with valproate exhibit varying risks for HBM. These observations suggest that physicians managing cases of young women with mood disorders should be diligent in their selection of antidepressant agents, especially when considering their combined use with valproate.

## Data availability statement

CZhu and CZho had full access to all the data and were responsible for the decision for submission for publication. All the data and supporting evidence (pictures and audio recordings) can be provided by CZhu and CZho.

## Ethics statement

The studies involving human participants were reviewed and approved by Ethics Committee of Tianjin Fourth Center Hospital. The patients/participants provided their written informed consent to participate in this study.

## Author contributions

CZhu, HT, and XS conceived and designed research. CZhu, GC, and CLin collected data, conducted research, and wrote the paper. JP, JZ, LW, SJ, CLiu, QZ, LY, QL, and CZho analyzed and interpreted data. All authors contributed to the article and approved the submitted version.

## Funding

This work was supported by grants from the National Natural Science Foundation of China (81871052 to CZhu and 81701326 to CZhu), the Tianjin Health Bureau Foundation (2014KR02 to CZhu), and the Key Projects of the Natural Science Foundation of Tianjin, China (17JCZDJC35700 to CZhu). The authors declare that this study received funding from Shandong Qilu Pharmaceutical Co., Ltd., Jiangsu Haosen Pharmaceutical Co., Ltd., Jiangsu Enhua Pharmaceutical Co., Ltd., Beijing Yimin Pharmaceutical Co., Ltd., Sinopharm Holding Medical Technology (Tianjin) Co., Ltd., and Beijing Jingdong Century Trading Co., Ltd. Uniform equivalent reported dosage was used to obviate exposure of the trade names. The funders were not involved in the study design, collection, analysis, interpretation of data, the writing of this article, and the decision to submit it for publication.

## Conflict of interest

The authors declare that the research was conducted in the absence of any commercial or financial relationships that could be construed as a potential conflict of interest.

## Publisher's note

All claims expressed in this article are solely those of the authors and do not necessarily represent those of their affiliated organizations, or those of the publisher, the editors and the reviewers. Any product that may be evaluated in this article, or claim that may be made by its manufacturer, is not guaranteed or endorsed by the publisher.
